# Chronic remote ischemic conditioning treatment in patients with chronic stable angina (EARLY-MYO-CSA): a randomized, controlled proof-of-concept trial

**DOI:** 10.1186/s12916-023-03041-z

**Published:** 2023-08-25

**Authors:** Quan Guo, Zhenzhou Zhao, Fan Yang, Zhiwen Zhang, Xiaoyu Rao, Jing Cui, Qingbo Shi, Kaiyuan Liu, Kang Zhao, Haiyu Tang, Liang Peng, Cao Ma, Jun Pu, Muwei Li

**Affiliations:** 1 Department of Cardiology, Department of Coronary Heart Disease of Central China Fuwai Hospital, Henan Key Laboratory for Coronary Heart Disease, Central China Fuwai of Zhengzhou University, Henan Provincial People’s Hospital, People’s Hospital of Zhengzhou University, No. 1 Fuwai Road, Zhengzhou, Henan Province China; 2 Department of Cardiology, School of Medicine, Ren Ji Hospital, Shanghai Jiao Tong University, 160 Pujian Road, Shanghai, 200127 China; 3 Medicine Department of Xizang, Minzu University, Xianyang, Shanxi China

**Keywords:** Chronic stable angina, Chronic remote ischemic conditioning, Coronary heart disease, Myocardial flow reserve

## Abstract

**Background:**

Chronic remote ischemic conditioning (CRIC) has been shown to improve myocardial ischemia in experimental animal studies; however, its effectiveness in patients with chronic stable angina (CSA) has not been investigated. We conducted a proof-of-concept study to investigate the efficacy and safety of a six-month CRIC treatment in patients with CSA.

**Methods:**

The EARLY-MYO-CSA trial was a prospective, randomized, controlled trial evaluating the CRIC treatment in patients with CSA with persistent angina pectoris despite receiving ≥ 3-month guideline-recommended optimal medical therapy. The CRIC and control groups received CRIC (at 200 mmHg) or sham CRIC (at 60 mmHg) intervention for 6 months, respectively. The primary endpoint was the 6-month change of myocardial flow reserve (MFR) on single-photon emission computed tomography. The secondary endpoints were changes in rest and stress myocardial blood flow (MBF), angina severity according to the Canadian Cardiovascular Society (CCS) classification, the Seattle Angina Questionnaire (SAQ), and a 6-min walk test (6-MWT).

**Results:**

Among 220 randomized CSA patients, 208 (105 in the CRIC group, and 103 in the control group) completed the treatment and endpoint assessments. The mean change in MFR was significantly greater in the CRIC group than in the control group (0.27 ± 0.38 vs. − 0.04 ± 0.25; *P* < 0.001). MFR increased from 1.33 ± 0.48 at baseline to 1.61 ± 0.53 (*P* < 0.001) in the CRIC group; however, a similar increase was not seen in the control group (1.35 ± 0.45 at baseline and 1.31 ± 0.44 at follow-up, *P* = 0.757). CRIC treatment, when compared with controls, demonstrated improvements in angina symptoms assessed by CCS classification (60.0% vs. 14.6%, *P* < 0.001), all SAQ dimensions scores (*P* < 0.001), and 6-MWT distances (440 [400–523] vs. 420 [330–475] m, *P* = 0.016). The incidence of major adverse cardiovascular events was similar between the groups.

**Conclusions:**

CSA patients benefit from 6-month CRIC treatment with improvements in MFR, angina symptoms, and exercise performance. This treatment is well-tolerated and can be recommended for symptom relief in this clinical population.

**Trial registration:**

[chictr.org.cn], identifier [ChiCTR2000038649].

**Supplementary Information:**

The online version contains supplementary material available at 10.1186/s12916-023-03041-z.

## Background

Chronic stable angina (CSA) is a common form of coronary heart disease (CHD) that is characterized by insufficient myocardial flow reserve (MFR) and a decline in quality of life. Besides pharmaceutical therapy, revascularization using percutaneous coronary intervention (PCI) or coronary artery bypass grafting (CABG) has been widely established for CSA patients [1]. However, both PCI and CABG are not suitable for some patients with symptomatic CSA with diffused distal coronary lesions. Due to limited measures for angina palliation in current clinical practice [2], a majority of CSA patients suffer from relevant symptoms and have a poor prognosis even if they receiving optimal medical treatment. Therefore, the development of novel treatments for these patients are urgently needed.

Remote ischemic conditioning (RIC) through periodic limb ischemia–reperfusion exposure confers protective effects in rendering remote tissues and organs resistant to ischemia–reperfusion injury [3, 4]. Previous studies identified the benefits of RIC in improving endothelial cell and coronary microcirculation function, increasing coronary blood flow, and myocardial ischemia tolerance [5–8]. A meta-analysis showed that a single session of RIC in patients with acute myocardial infarction (AMI) undergoing primary PCI significantly reduce myocardial infarct size compared with the controls [9]. Moreover, improvements in perfusion pressure and collateral circulatory flow in the distal coronary arteries has been reported. However, a single short-term RIC (four cycles of 5-min upper arm ischemia and reperfusion) failed to improve resting myocardial blood flow (MBF) in patients with suspected ischemic coronary artery disease [10]. Emerging data suggests that repeated daily episodes of limb RIC, termed chronic remote ischemic conditioning (CRIC), may have beneficial effects greater than those conferred by a single RIC stimulus [11–13]. Basic studies have demonstrated that CRIC could promote angiogenesis in ischemic tissue, enhance the function of vascular endothelial and collateral circulation, and improve blood perfusion [14–16]. In addition, an observational study involving patients with chronic heart failure showed that repeated RIC for 1 week increased the coronary flow reserve [17]. To date, no studies have conducted on the use of CRIC in patients with CSA. Therefore, we hypothesized that a long-term uninterrupted RIC would improve the MFR in patients with CSA, resulting in angina symptom relief, and aimed to test this hypothesis in a prospective, randomized, sham-control proof-of-concept trial.

## Methods

### Trial design

The trial followed a prospective, randomized, sham-control design. It complied with the Declaration of Helsinki and was approved by the New Business and Technology Ethics Committee of Fuwai Central China Cardiovascular Hospital, Zhengzhou University (Zhengzhou, China). We followed the Consolidated Standards of Reporting Trials (CONSORT) checklist [18] to report this study (Additional file 1: CONSORT checklist). All participants signed an informed consent form. This trial was registered with the Chinese Clinical Trial Registry (ChiCTR2000038649).

### Participants

Eligible participants were those (1) > 18 years old, (2) having CSA confirmed by angiography without complete revascularization (> 50% stenosis on angiography and the quantitative flow ratio < 0.75 for at least one main coronary artery or branches), and (3) with persistent angina pectoris after receiving ≥ 3-month guideline-recommended optimal medical therapy (the main drugs include β-blocker, calcium channel blocker (CCB), nitrates, ivabradine, trimetazidine, nicorandil, antiplatelet agents, angiotensin-converting enzyme inhibitor (ACEI) or angiotensin receptor blocker (ARB), and statin; all enrolled patients received the recommended dose in accordance with the guidelines, unless contraindicated). Exclusion criteria included the following: intolerance to RIC therapy; stenosis of the left main stem ≥ 50%; pregnancy or having a plan for it; a history of arterial or venous thrombosis in upper limbs; comorbidities of severe valvular disease, congenital heart disease, severe arrhythmia, aortic dissection, aortic aneurysm, cardiomyopathy, and severe uncontrolled hypertension (defined as systolic blood pressure > 180 mmHg and/or diastolic blood pressure > 110 mmHg after taking medication); life expectancy < 1 year; malignant tumor; poor compliance to RIC; patients with acute myocardial infarction or coronary revascularization in the past 1 year; use of glibenclamide [19]; and MFR ≥ 2.5 as determined using single photon emission computed tomography (SPECT).

### RIC tolerance assessment

Before randomization, each patient underwent an RIC tolerance evaluation. RIC intolerances include (1) unbearable limb pain and numbness during cuff inflation and (2) unable to sustain 35 consecutive minutes of restricted activity by RIC device. Patients with RIC intolerance will be excluded.

### Randomization

**Fig. 1:**
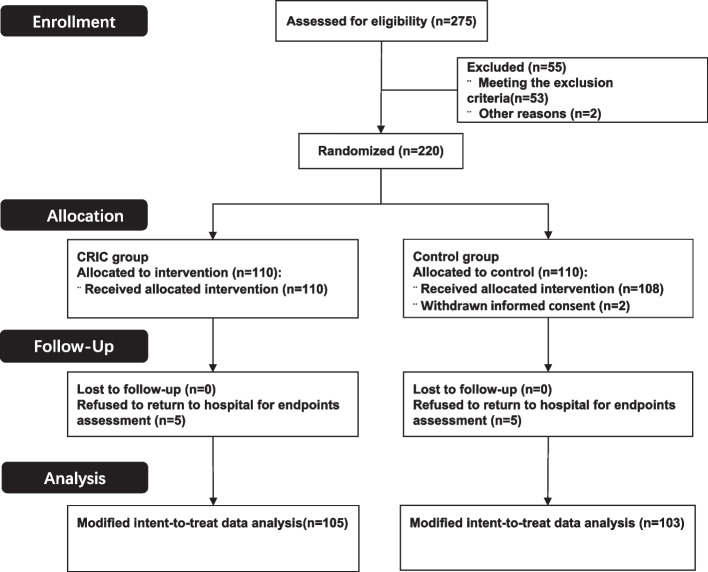
CONSORT flow diagram for the study

From October 2020 to May 2022, 275 patients with CSA who were referred to Fuwai Central China Hospital were recruited. Of these, 220 were randomized after screening according to the inclusion and exclusion criteria. Among them, two withdrew their informed consent after randomization, and 10 participants refused to return to hospital for endpoints assessment (Fig. 1). A total of 208 patients (105 in the CRIC group and 103 in the control group) completed the treatment and endpoints assessments. Randomization (1:1 ratio) was performed by an independent statistician using a computer-generated list and with stratifications by factors of sex and age ≥ 50 years.

### Implementation of treatment and follow-up

All patients received standard optimal medical therapy according to current guidelines. For those assigned to the CRIC group, a semi-automatic RIC device with a controller and a cuff (GTHR Medical Technology company, Shenzhen, China) was used. The device has been approved by the Food and Drug Administration in Guangdong Province, China. Once the cuff was attached to the participant’s upper arm and the controller was turned on, the device automatically performed the RIC procedure. Each RIC procedure took 35 min with four cycles of cuff inflation at 200 mmHg pressure (5 min for each cycle) and 5 min intervals of relaxation between two cycles. This procedure was performed twice per day. Patients in the control group received sham RIC using a device with the identical appearance to the RIC device at a cuff pressure of 60 mmHg. One investigator held a device-use training during the trial screening phase to ensure that all participants could operate the device correctly. During follow-up, participants were asked to provide device usage records. Participants were evaluated by clinic visit or telephone at 1 and 3 months after randomization and were asked to return for face-to-face assessments at 6 months.

### Primary and secondary study endpoints

The primary endpoint of this trial was the change of MFR at 6 months (± 14 days) post-randomization. Secondary endpoints included 6-month changes of rest and stress MBF, Canadian Cardiovascular Society (CCS) classification-defined angina improvement, all dimensions of the Seattle Angina Questionnaire (SAQ) score, and the distance from 6-min walk test (6-MWT).

### SPECT assessment

All patients underwent MBF quantification using SPECT at the Central China Fuwai Hospital at baseline and at 6 months (± 14 days) post-randomization. SPECT was performed with a cadmium zinc telluride camera system (Discovery NM 530c; GE, Boston, MA) using a 2-day protocol. On day 1, the patient underwent low-dose computerized tomography (CT) scanning for physical image processing correction and was administered an adenosine injection (injection rate of 0.14 mg/kg/min) after drinking 500 ml of water. The load list mode dynamic SPECT data collection started at 2 min and 50 s. At the third minute, 20 mCi 99mTc-MIBI was injected as an intravenous bolus, and dynamic SPECT data acquisition continued for 10 min. On day 2, the patient started resting list-mode dynamic SPECT data acquisition after drinking 500 ml of water; 10 s later, 20 mCi 99mTc-MIBI myocardial imaging agent was injected as an intravenous bolus, and dynamic SPECT data acquisition continued for 10 min. The corresponding left ventricle (LV) MBF quantification data were analyzed using the MyoFlowQ software [20]. The MFR was calculated as the ratio of stress to rest MBF values. According to the SPECT flow status, a polar map was converted from the rest MBF, stress MBF, and MFR using the flow diagram [21, 22]. The left ventricle myocardial blood flow restriction of each patient was classified into seven degrees: definitely normal, normal limit, mildly abnormal, moderately abnormal, ischemia, steal, and infarct. Then, the flow restriction extent was calculated as a percentage using the MyoFlowQ software. During this study, we identified the sum of the percentages of ischemia and steal flow status on the polar map as the reversible myocardial ischemia extent (RMIE) in order to more intuitively observe the improvement of myocardial ischemia (Additional file 2: Figure S1). The SPECT results were assessed by three nuclear medicine readers who were blinded to the randomization and participants’ clinical information using MyoFlowQ software and then the mean of MBF quantification was calculated. To assess the reproducibility of SPECT, intra-observer and inter-observer variability was validated in SPECT results from all 208 participants. The first nuclear medicine reader conducted a repeat MBF quantification analysis after an interval of 3 months for intra-observer variability. Inter-observer variability was performed using the results obtained by the second nuclear medicine reader who had participated in the initial analysis.

### Angina pectoris assessment

Any change in angina symptoms was assessed using the CCS classification, SAQ score, and 6-MWT by investigators blinded to the randomization and participants’ clinical information.

The CCS classification was used to assess the severity of angina pectoris based on the combination of physician’ s assessment and patient’ s self-reported symptoms. The SAQ is a 19-item questionnaire that measures five domains related to angina pectoris: angina frequency, angina stability, physical limitation, treatment satisfaction, and disease perception [23]. Each domain has a score from 0 to 100, with a higher score representing an improvement of angina pectoris. More information on CCS classification and SAQ is available in the Additional file 3. The 6-MWT was performed as follows: after resting for 5–10 min, the patient walked up and down a 30 m straight corridor for 6 min; the assessor encouraged the patient to walk as fast as possible while following directly behind; the assessor recorded the maximal distance covered by the patient in 6 min or < 6 min if the patient stopped earlier.

### Safety assessment

We evaluated the safety of CRIC treatment by assessing major adverse cardiovascular events and local adverse reactions to RIC in the limbs. Major adverse cardiovascular events included all-cause death, nonfatal myocardial infarction, heart failure, and stroke after randomization. Detailed definitions of events are provided in our previous studies [24, 25]. Local limb adverse reactions reported by participants or investigators, included skin ecchymosis, limb pain, limb weakness, and limb arteriovenous thrombosis. All adverse events were judged by an independent committee.

### Sample size

The sample size of the present trial was calculated based on the findings of our pilot study: there was a 0.20 difference in the 6-month MRF change from baseline across two randomized groups, with 0.15 ± 0.46 in the CRIC group and − 0.05 ± 0.41 in sham the CRIC group. Accordingly, group sample sizes of 102 per group were required to achieve 90% power to detect a difference of 0.20 in a two-sample *t*-test allowing unequal variance study design. The standard deviation of CRIC and sham CRIC was 0.46 and 0.41, respectively, while the alpha level was two-sided 0.05. With an expected 5% dropout rate, at least 214 patients should be enrolled. Power calculation was performed using the PASS software (version 15; NCSS, LLC, Kaysville, Utah, USA). More information on sample size calculation is available in Additional file 3.

### Statistical analysis

The primary analysis was performed using the full analysis set on a modified intention-to-treat basis. Statistical analysis was performed using SPSS software (version 25.0; SPSS Inc., Armonk, NY). Categorical variables were presented as the number of cases and percentages. Comparisons between groups were performed using the chi-square test. Continuous variable data that conformed to normal distributions were presented as the mean ± standard deviation. Comparisons between groups were performed using the unpaired Student’s *t*-test. For repeated-measures data, an analysis of within-group measurements was performed using the paired *t*-test. Continuous variables that did not conform to normal distributions are expressed as median (interquartile ranges). Between-group comparison were processed using the Mann–Whitney *U* test. Repeated-measures data were processed using the Wilcoxon two-sample test. The Bland–Altman analysis, intraclass correlation coefficients (ICC), and coefficient of variation (COV) were used to evaluate the inter and intra-observer variability of MBF, MFR, and RMIE. *P* < 0.05 was considered statistically significant (two-sided). Both the primary and secondary endpoint indicators of this study relate to outcomes at follow-up, while data on any endpoint indicators were not available for patients lost to follow-up after randomization, and the data for the remaining patients analyzed met the modified intention-to-treat data set criteria.

## Results

### Population characteristics

In total, 208 patients completed the study consisting of 137 (65.9%) men and with a mean age of 61.99 ± 10.53 years. Among the participants, 44 (21.2%) had a history of myocardial infarction, 133 (63.9%) had undergone PCI, and 9 (4.3%) had undergone CABG. Participants in both groups had high syntax scores (22.33 ± 7.52), suggesting the complexity of their coronary lesions. The baseline characteristics were comparable between the two randomized groups (Table 1).

**Table 1:** Baseline characteristics of the participants

		CRIC (*n* = 105)	Control (*n* = 103)	*P*
Age (years)		61.12 ± 10.85	62.86 ± 10.15	0.234
Male [*n* (%)]		68 (64.8)	69 (67.0)	0.735
Smoking [*n* (%)]		26 (24.8)	27(26.2)	0.810
Hypertension [*n* (%)]		45(42.9)	44 (42.7)	0.984
Diabetes [*n* (%)]		36 (34.3)	39 (37.9)	0.591
Hyperlipidemia [*n* (%)]		47 (44.8)	46 (44.7)	0.988
PCI history [*n* (%)]		43 (41.0)	32(31.1)	0.138
CABG history [*n* (%)]		4(3.8)	5(4.9)	0.747
MI history [*n* (%)]		20(19.0)	24(23.3)	0.453
BMI		25.65 ± 2.84	25.54 ± 3.61	0.808
Syntax score		22.27 ± 7.01	22.39 ± 8.05	0.908
LVEF (%)		58.66 ± 11.51	55.82 ± 12.55	0.090
NYHA classification [*n* (%)]	I	58 (55.2)	52 (50.5)	0.747
	II	36 (34.3)	42 (40.8)	
	III	11 (10.5)	9 (8.7)	
Numbers of vessels with critical stenosis [*n* (%)]	1	54 (51.4)	50 (48.5)	0.689
	2	45 (42.9)	44 (42.7)	
	3	6 (5.7)	9 (8.7)	
Medications [*n* (%)]	Aspirin	105 (100)	103 (100)	-
	Statin	102 (97.1)	101 (98.1)	0.509
	β-blocker	95 (90.5)	95 (92.2)	0.652
	Ivabradine	9(8.6)	6(5.8)	0.444
	ACEI/ARB	70 (66.7)	61 (59.2)	0.266
	Long-acting nitrate	100 (95.2)	98 (95.1)	0.614
	CCB	42 (40.0)	39 (37.9)	0.752
	Trimetazidine	94 (89.5)	95 (92.2)	0.498
	Nicorandil	49 (46.7)	43 (41.7)	0.475
	Metformin	28 (26.7)	20 (19.4)	0.215
	SGLT2i	39 (37.1)	38 (36.9)	0.970
	Insulin	16 (15.2)	13 (12.6)	0.586
	PCSK9i	42 (40.0)	35 (34.0)	0.369

*CRIC* Chronic remote ischemic conditioning, *PCI* Percutaneous coronary intervention, *BMI* Body mass index, *LVEF* Left ventricular ejection fraction, *NYHA* New York Heart Association, *ACEI/ARB* Angiotensin-converting enzyme inhibitor/angiotensin receptor blocker, *CCB* Calcium channel blocker, *SGLT2i* Sodium-glucose cotransporter 2 inhibitor, *PCSK9i* Proprotein convertase subtilisin/kexin type 9 inhibitor

### SPECT outcomes

**Fig. 2:**
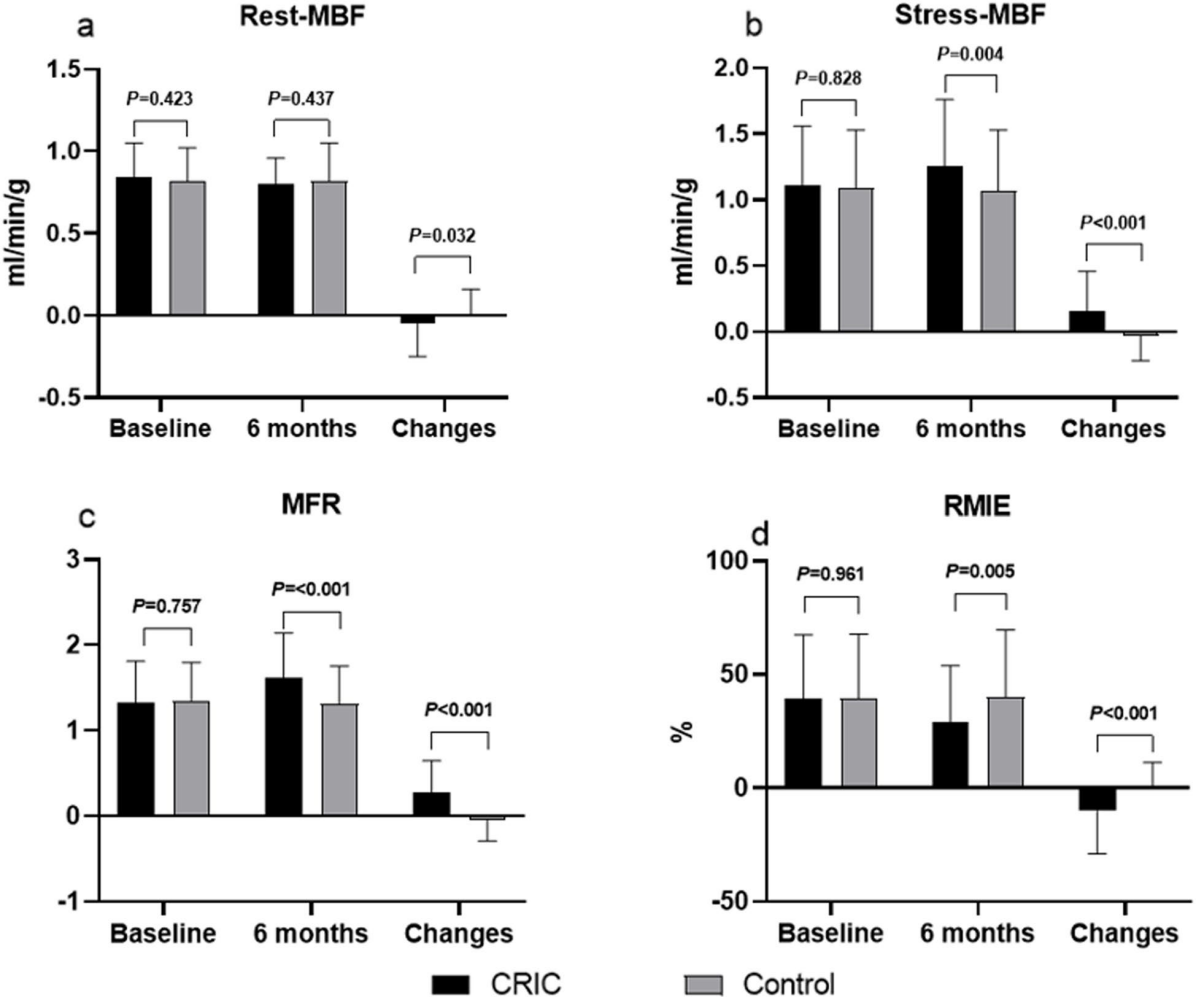
Comparison of myocardial flow outcomes at baseline and the 6-month follow-up evaluation. **a** There was no significant difference in the rest myocardial blood flow (MBF) between groups at baseline and 6 months; however, a significant change from baseline at 6 months was observed in the chronic remote ischemic conditioning (CRIC) group. **b** There was no significant difference in stress MBF between the groups at baseline; however, at 6 months, the CRIC group had significantly higher stress MBF and a greater change from baseline than the control group. **c** There was no significant difference in myocardial flow reserve (MFR) between groups at baseline; however, the CRIC group had a significantly higher MFR and greater change from baseline than the control group at 6 months. **d** There was no significant difference in reversible myocardial ischemia extent (RMIE) between the groups at baseline; however, the RMIE was significantly lower in the CRIC group than in the control group at 6 months. The CRIC group showed a greater change from baseline than the control group at 6 months

SPECT outcomes showed that both groups had low global rest and stress MBF values at baseline (rest: 0.84 ± 0.21 vs. 0.82 ± 0.20 mL/min/g, *P* = 0.423; stress: 1.11 ± 0.45 vs. 1.09 ± 0.44 mL/min/g, *P* = 0.828). After 6 months, the control group had no significant changes in rest and stress MBF measurements. Conversely, rest MBF values significantly decreased in the CRIC group at 6 months compared with baseline (0.80 ± 0.16 mL/min/g vs. 0.84 ± 0.21 mL/min/g; *P* = 0.020). Stress MBF in the CRIC group significantly increased in 6 months compared with baseline (1.26 ± 0.50 mL/min/g vs. 1.11 ± 0.45 mL/min/g; *P* < 0.001). The MFR increased from baseline at 6-month follow-up in the CRIC group (1.33 ± 0.48 vs. 1.61 ± 0.53; *P* < 0.001) but not in the control group (1.35 ± 0.45 vs. 1.31 ± 0.44; *P* = 0.608). The mean 6-month change of MFR was significantly greater in the CRIC group than in the control group (0.27 ± 0.38 vs. − 0.04 ± 0.25; *P* < 0.001). The RMIE significantly decreased from baseline at 6-months follow-up in the CRIC group (39.42 ± 28.15% vs. 29.33 ± 24.66%; *P* < 0.001), but not in the control group (39.61 ± 28.14 vs. 40.16 ± 29.47; *P* = 0.108; Table 2, Fig. 2).

**Table 2:** SPECT outcomes

	Time	CRIC (*n* = 105)	Control (*n* = 103)	*P*
MFR	Baseline	1.33 ± 0.48	1.35 ± 0.45	0.757
	Follow-up	1.61 ± 0.53	1.31 ± 0.44	< 0.001
	P*	< 0.001	0.608	
	Δ	0.27 ± 0.38	− 0.04 ± 0.25	< 0.001#
Rest MBF (ml/min/g)	Baseline	0.84 ± 0.21	0.82 ± 0.20	0.423
	Follow-up	0.80 ± 0.16	0.82 ± 0.23	0.437
	P*	0.020	0.872	
	Δ	− 0.05 ± 0.20	0.00 ± 0.16	0.032#
Stress MBF (ml/min/g)	Baseline	1.11 ± 0.45	1.09 ± 0.44	0.828
	Follow-up	1.26 ± 0.50	1.07 ± 0.46	0.004
	P*	< 0.001	0.147	
	Δ	0.15 ± 0.31	− 0.03 ± 0.19	< 0.001#
RMIE (%)	Baseline	39.42 ± 28.15	39.61 ± 28.14	0.961
	Follow-up	29.33 ± 24.66	40.16 ± 29.47	0.005
	P*	< 0.001	0.108	
	Δ	− 10.09 ± 19.01	0.54 ± 10.74	< 0.001#

*CRIC* Chronic remote ischemic conditioning, *MFR* Myocardial flow reserve, *MBF* Myocardial blood flow, *RMIE* Reversible myocardial ischemia extent, Δ represents the difference between the follow-up and baseline

^*^Paired *t*-tests were used to compare baseline and follow-up

^#^Mann–Whitney *U* test

### Reproducibility of SPECT outcomes

Intra-observer variability yielded good concordance for MBF (bias =  − 0.02 to 0.01, ICC = 0.86 to 0.98, and COV = 8.04 to 13.67%), MFR (bias =  − 0.02 to 0.00, ICC = 0.90 to 0.92, and COV = 14.26 to 16.17%), and RMIE (bias =  − 0.36 to − 0.32, ICC = 0.98 to 0.9, and COV = 10.57 to 12.11%). The ICC showed moderate inter-observer agreement in the rest MBF of baseline (ICC = 0.74) and good in other MBF assessment (ICC = 0.88–0.95), MFR (ICC = 0.87 to 0.89), and RMIE (ICC = 0.99). The Bland–Altman analysis and COV showed good inter-observer agreement for MBF (bias =  − 0.02 to 0.02 and COV = 12.67 to 18.18%), MFR (bias =  − 0.03 to 0.01 and COV = 16.77 to 18.59%), and RMIE (bias =  − 0.50 to − 0.26 and COV = 10.86 to 11.73%; Additional file 4: Figure S2, Additional file 5: Table S1).

### Angina pectoris assessment

**Fig. 3:**
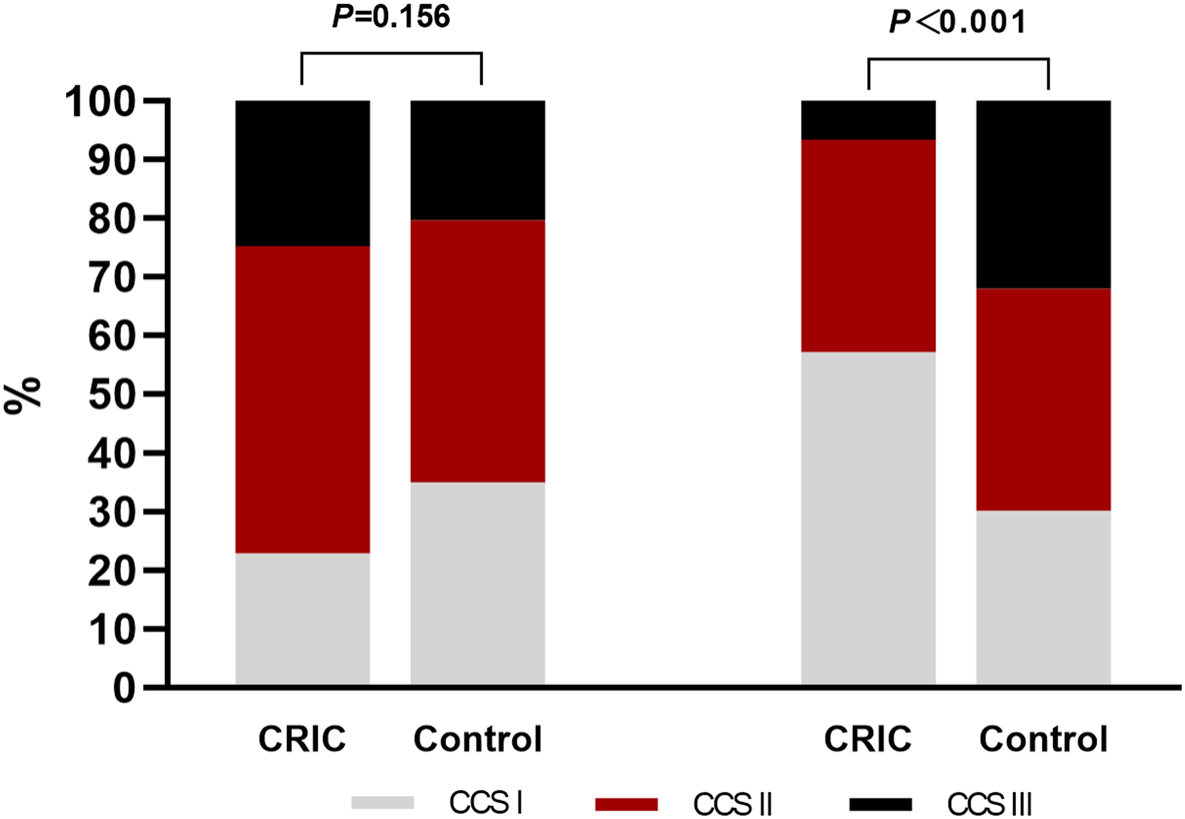
The chronic remote ischemic conditioning (CRIC) showed a significant overall improvement in self-reported symptoms according to the Canadian Cardiovascular Society (CCS) with a change in the composition ratio of the different classifications at the time of follow-up

A significant difference in terms of 6-month angina symptoms relief according to the CCS classification between groups was observed, with a higher proportion of patients having symptom improvement in the CRIC group than in the control group (60% vs. 14.6%, *P* < 0.001; Table 3, Fig. 3).

**Table 3:** CCS classification

CCS classification [*n* (%)]	Time		CRIC (*n* = 105)	Control (*n* = 103)	*P*
	Baseline	I	24 (22.9)	36 (35.0)	0.156
		II	55 (52.4)	46 (44.7)	
		III	26 (24.8)	21 (20.4)	
	Follow-up	I	60 (57.1)	31 (30.1)	< 0.001
		II	38 (36.2)	39 (37.9)	
		III	7 (6.7)	33 (32.0)	
CCS classification changed [*n* (%)]	No change		27 (25.7)	60 (58.3)	< 0.001
	Improved		63 (60.0)	15 (14.6)	
	Deteriorated		15 (14.3)	28 (27.2)	

*CCS* Canadian Cardiovascular Society, *CRIC* Chronic remote ischemic conditioning

**Fig. 4:**
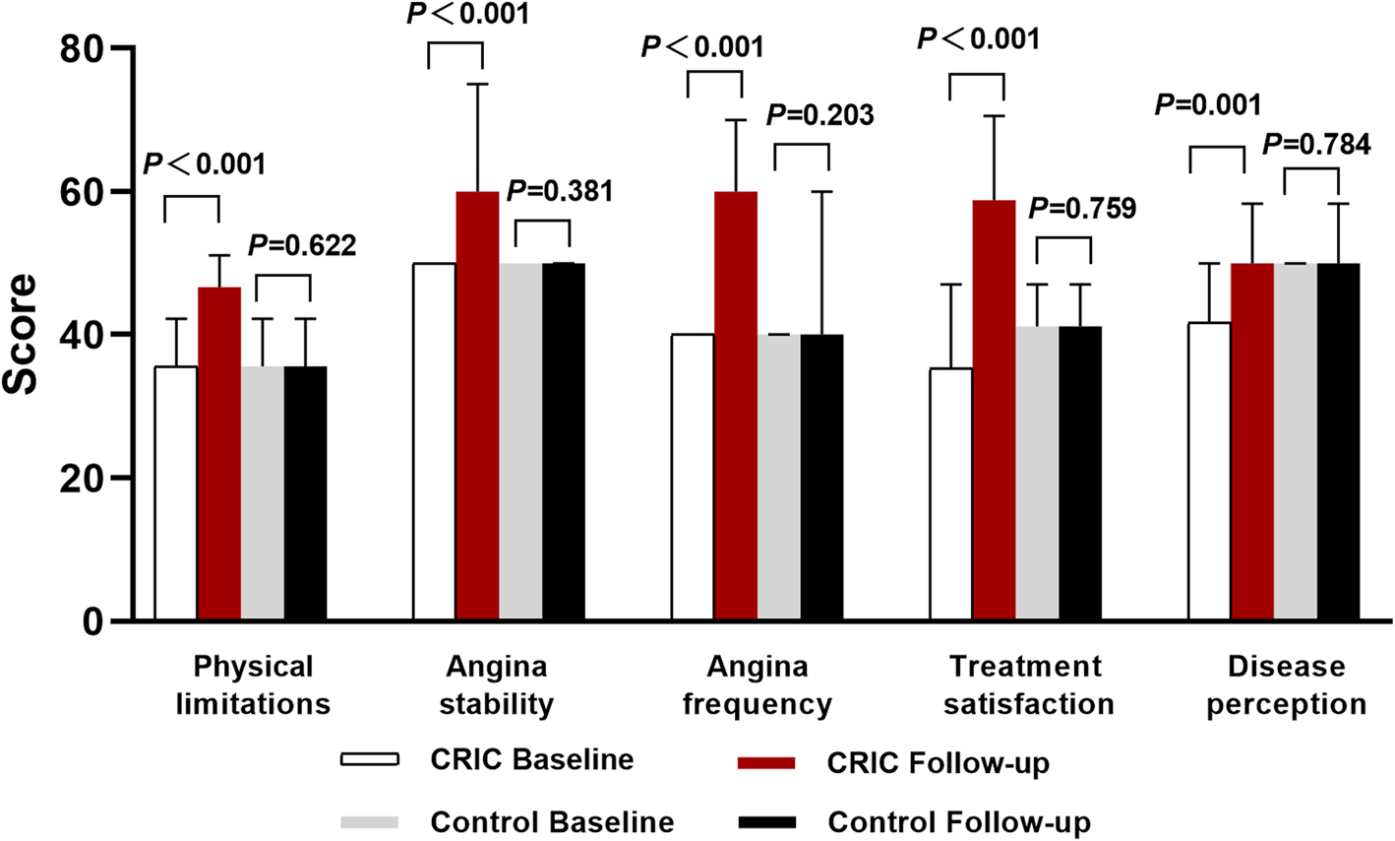
Changes in the five dimensions of the Seattle Angina Questionnaire (SAQ) score at baseline and follow-up. The chronic remote ischemic conditioning (CRIC) group patients had improved scores in all five dimensions

The five dimensions of SAQ were not significantly different between the two groups at baseline; however, all the dimension scores were significantly higher in the CRIC group than in the control group, with the exception of disease perception score (*P* = 0.126). Moreover, disease perception scores significantly increased from baseline in the CRIC group at 6-month follow-up (*P* = 0.001; Table 4, Fig. 4).

**Table 4:** Seattle Angina Questionnaire score

	Time	CRIC (*n* = 105)	Control (*n* = 103)	*P*
Physical limitation	Baseline	35.56 (33.33 ~ 42.22)	35.56 (33.33 ~ 42.22)	0.869
	Follow-up	46.67 (43.33 ~ 51.11)	35.56 (33.33 ~ 42.22)	< 0.001
	P*	< 0.001	0.622	
Anginal stability	Baseline	50.00 (50.00 ~ 50.00)	50.00 (50.00 ~ 50.00)	0.855
	Follow-up	60.00 (50.00 ~ 75.00)	50.00 (40.00 ~ 50.00)	< 0.001
	P*	< 0.001	0.381	
Angina frequency	Baseline	40.00 (30.00 ~ 40.00)	40.00 (30.00 ~ 40.00)	0.208
	Follow-up	60.00 (60.00 ~ 70.00)	40.00 (30.00 ~ 60.00)	< 0.001
	P*	< 0.001	0.203	
Treatment satisfaction	Baseline	35.29 (29.41 ~ 47.06)	41.18 (29.41 ~ 47.06)	0.249
	Follow-up	58.82 (47.06 ~ 70.59)	41.18 (35.29 ~ 47.06)	< 0.001
	< 0.001	< 0.001	0.759	
Disease perception	Baseline	41.67 (41.67 ~ 50.00)	50.00 (41.67 ~ 50.00)	0.142
	Follow-up	50.00 (41.67 ~ 58.33)	50.00 (41.67 ~ 58.33)	0.126
	P*	0.001	0.784	

*CRIC* Chronic remote ischemic conditioning

^*^Paired analyses were performed using the Wilcoxon two-sample test

At baseline, the 6-MWT results were comparable between the two groups (400 [320–450] vs. 405 [330–470] m; *P* = 0.193]. After 6 months, the distance covered over 6 min was greater among patients in the CRIC group than those in the control group (440 [400–523] vs. 420.00 [330–475] m; *P* = 0.016; Table 5).

**Table 5:** Six-minute walk test

	Time	CRIC (*n* = 105)	Control (*n* = 103)	*P*
6-min walk test(m)	Baseline	400 (320 ~ 450)	405 (330 ~ 470)	0.193
	Follow-up	440 (400 ~ 523)	420 (330 ~ 475)	0.016
	P*	< 0.001	0.106	

*CRIC* Chronic remote ischemic conditioning

^*^Paired analyses were performed using the Wilcoxon two-sample test

### Changes in medical interventions

During the study period, three patients in the CRIC group and four patients in the control group underwent PCI (2.9% vs. 2.3%, *P* = 0.720). After 6 months, a greater proportion of patients in the CRIC group exhibited a decrease in the frequency of short-acting nitrate administration (70.5% vs. 15.5%, *P* < 0.001; Table 6).

**Table 6:** Changes in medical interventions during the study period

Intervention	CRIC (*n* = 105)	Control (*n* = 103)	*P*
Revascularization [*n* (%)]			
Percutaneous coronary intervention	3 (2.9)	4 (3.9)	0.720
Coronary artery bypass graft	0	0	-
Frequency of short-acting nitrates [*n* (%)]			
Decrease	74 (70.5)	16 (15.5)	< 0.001
No-change	28 (26.7)	72 (69.9)	
Increase	3 (2.9)	15 (14.6)	

*CRIC* Chronic remote ischemic conditioning

### Safety assessment

No cardiovascular death or heart failure events occurred in either group during the 6-month follow-up period. A few participants experienced nonfatal myocardial infarction (3 [2.9%] in the CRIC group vs. 2 [1.9%] in the control group; *P* = 0.509). All participants in the CRIC group tolerated RIC well. A few participants reported transient hand numbness or sweating while using the RIC device; however, complete relief was achieved immediately after the procedure. The presence of skin ecchymosis or upper extremity pain was slightly higher in the CRIC group than in the control group; however, the difference was no statistical significance (*P* = 0.117 and *P* = 0.351, respectively). Furthermore, neither group experienced arteriovenous thrombosis events during the trial (Table 7).

**Table 7:** Safety analyses

	CRIC (*n* = 105)	Control (*n* = 103)	*P*
Major adverse cardiovascular events [*n* (%)]			
Death	0	0	-
Nonfatal myocardial infarction	3 (2.9)	2 (1.9)	0.509
New-onset heart failure	0	0	-
Stoke	0	0	
Local response [*n* (%)]			
Skin ecchymosis	12 (11.4)	6 (5.8)	0.117
Limb pain	4 (3.8)	2 (1.9)	0.351
Limb weakness	3 (2.9)	4 (3.9)	0.720
Limb arteriovenous thrombosis	0	0	-
Hand numbness	16 (15.2)	7 (6.8)	0.052
Hand sweating	13 (12.4)	8 (7.8)	0.269

*CRIC* Chronic remote ischemic conditioning

## Discussion

This randomized, proof-of-concept study investigated the clinical efficacy and safety of CRIC in CSA patients and found that uninterrupted CRIC treatment twice daily for 6 months was well-tolerated by all patients and improved MFR and angina symptoms with assured safety. Thus, this novel study provides the first evidence to support the therapeutic potential of CRIC as a low-cost and non-invasive intervention strategy for symptomatic CSA treatment.

Although animal studies have revealed underlying mechanisms of RIC in improving microcirculation, results from clinical studies have been inconsistent regarding the effects of RIC on target organ protection in different diseases [26–28]. Although a single RIC session was shown to reduce myocardial infarct size in patients with AMI undergoing primary PCI, it did not improve clinical outcomes [29]. Similarly, a single RIC session failed to improve clinical outcomes and the incidence of acute kidney injury in patients undergoing cardiac surgery [30]. Perhaps, these findings can be attributed to insufficient dosing where by the frequency of RCI administered was too low to induce any effects [31]. However, another study revealed that increased limb ischemia–reperfusion cycles or ischemia intervals within one RIC procedure did not work [32]. Daily repeated RIC was successfully implemented to increase the dose and potential enhancement effect in some animal and clinical studies. Khan et al. [14] showed that ≥ 1-month uninterrupted daily RIC could promote cerebrovascular remodeling in a mouse model of vascular dementia; Ding et al. [15] demonstrated that repeated RIC for 2 years could improve cerebral blood perfusion in patients with moyamoya disease. The RICA trial reported that a 12-month RIC reduced the incidence of ischemic stroke in the RIC group with good compliance [33], though the inter-group difference was insignificant. However, no previous studies have investigated the effects of long-term RIC on MFR in patients with chronic myocardial ischemia. Our results of this study showed that long-term uninterrupted RIC improved MFR by increasing stress MBF and decreasing rest MBF.

MFR deficiency is the main cause of angina during daily activities in patients with CSA. When epicardial vessel stenosis persists, stress MBF is mainly influenced by microcirculatory resistance [34]. RIC can increase stress MBF by either reducing microvascular resistance or improving collateral circulation function [5, 35, 36]. Patients with CHD risk factors often have impaired endothelium-dependent diastolic function, resulting in the abnormal regulation of vasomotor function in resistance artery [37, 38]. RIC stimulates the release of anti-inflammatory mediators to improve vasomotor and vascular endothelial function, thus ameliorating high microcirculatory resistance is ameliorated [5, 39–41]. Sufficient collateral circulation also contributes to an increased in stress MBF. RIC improves collateral circulation by enhancing the endothelial function of the existing small vessel network and promoting angiogenesis. Ischemia and hypoxia, the fundamental stimuli of angiogenesis [42], can be induced by RIC in limb tissue, resulting in release more proangiogenic substances and travel to the myocardial ischemic tissue. This mechanism has been confirmed in animal experiments on angiogenesis using plasma isolated from individuals who received RIC [16, 43]. In addition, RIC-mediated improvement if small vessel endothelial function also contributes to the vessel remodeling of delicate newly generated small vessels into larger, muscularized functional collateral vessels [44].

Consistent with previous research, CRIC decrease MBF at rest [10, 17], which may have been attributed to autonomic nervous system regulation [45, 46], whereby RIC reduced cardiac workload by suppressing sympathetic excitability, ultimately leading to a reduction in myocardial oxygen consumption.

The results of the present study verified the improvement in angina symptoms by CRIC. Although there were only small increases in the absolute change in MFR at 6 months in the CRIC group, the mean improvement from baseline was approximately 20%, and the RMIE decreased by approximately 10%. These changes were sufficient to induce an improvement in angina symptoms.

In addition to its impact on MFR, CRIC has been found to stimulate adenosine triphosphate-sensitive potassium channels, facilitate the expression of anti-ischemic factors such as protein kinase A and stress proteins, and augment myocardial resistance to hypoxia [47, 48]. These effects have also been linked to the alleviation of angina symptoms.

In this study, not all patients in the CRIC group had improved MFR and angina symptoms: 19 patients in the CRIC group had a decrease in MFR. Several reasons may contribute this phenomenon: (1) patients experienced rapid progression of epicardial stenosis, resulting in further reduction in blood flow. Note that 15 patients in the CRIC group in our study had an increase in CCS classification during 6-month follow-up. (2) The participants included some diabetic patients, and peripheral neuropathy due to diabetic complications may lead to nociceptive insensitivity, which can result in attenuating the effect of RIC [49, 50].

## Study limitations

First, it was a single-center study, and selection bias cannot be fully avoided. Second, as a proof-of-concept trial, our principal aim was to investigated the efficacy and safety of a 6-month CRIC treatment among CSA patients. Thus, surrogate endpoint (i.e., the 6-month change of MFR) was chosen. Third, whether a long-term uninterrupted RIC is beneficial to the clinical prognosis is unknown. Our sample size was not enough to evaluate the effect of RIC on clinical cardiovascular outcomes. Further large-scale, multicenter trials with clinical outcomes as study endpoint are required. Fourth, the measurement of MFR in our study was assessed using SPECT. While positron emission tomography (PET) is generally considered to be more accurate and repeatable than SPECT [51], it is noteworthy that all SPECT scans in this study were conducted in a core laboratory and analyzed using MyoFlowQ software with corrections [52, 53], which demonstrated strong intra- and inter-observer consistency (Additional file 5: Table S1). Lastly, the mechanisms of limb RIC to protect remote organs are complex and involve a variety of traditional signaling pathways [54], which warrant more work.

## Conclusions

This study demonstrated the efficacy and safety of a 6-month CRIC to improving MFR, angina symptoms, and exercise performance capacity. This effect may be induced by improving coronary microcirculation, reducing resting myocardial oxygen consumption, and enhancing myocardial resistance to an ischemia and hypoxia conditions. CRIC may be an effective, noninvasive, and cost-saving option for symptom relief in managing CSA.

### Supplementary Information

**Additional file 1. **CONSORT checklist.

**Additional file 2: Figure S1. **An example of the myocardial blood flow (MBF) quantification changes presented by single-photon emission computed tomography (SPECT) before and after treatment in a chronic remote ischemic conditioning (CRIC) group patient. Part A: Blood flow status polar map, with a bar graph to represent the extent for each flow status; Part B: The blood flow diagram; Part C: Quantification of rest MBF and stress MBF and myocardial flow reserve (MFR) globally and in individual parts of the left ventricle.

**Additional file 3. **Supplementary methods included Angina pectoris assessment and Power analysis equation.

**Additional file 4: Figure S2. **Differences in repeated measurements of single-photon emission computed tomography outcomes for intra-observer analysis (A to H) and inter-observer analysis (I to P). The dashed lines indicate 95% confidence limits. The results show good agreement across all indicators.

**Additional file 5: Table S1. **Intra- and Inter-observer variability in SPECT outcomes.

## Data Availability

The datasets generated and analyzed during the current study are not publicly available because of patient privacy but are available from the corresponding author upon reasonable request.

## Abbreviations

6-MWT: 6-Minute walk test
CCS: Canadian Cardiovascular Society
CSA: Chronic stable angina
CRIC: Chronic remote ischemic conditioning
CABG: Coronary artery bypass grafting
CHD: Coronary heart disease
COV: Coefficient of variation
ICC: Interclass correlation coefficient
MACEs: Major adverse cardiovascular events
MBF: Myocardial blood flow
MFR: Myocardial flow reserve
PCI: Percutaneous coronary intervention
RIC: Remote ischemic conditioning
RMIE: Reversible myocardial ischemia extent
SAQ: Seattle Angina Questionnaire
SPECT: Single-photon emission computed tomography
