# Supplementary figures and images for: Chronic remote ischemic conditioning treatment in patients with chronic stable angina (EARLY-MYO-CSA): a randomized, controlled proof-of-concept trial
Source: BMC Med. 2023 Aug 25;21:324. doi: 10.1186/s12916-023-03041-z (PMC10463998; doi:10.1186/s12916-023-03041-z)

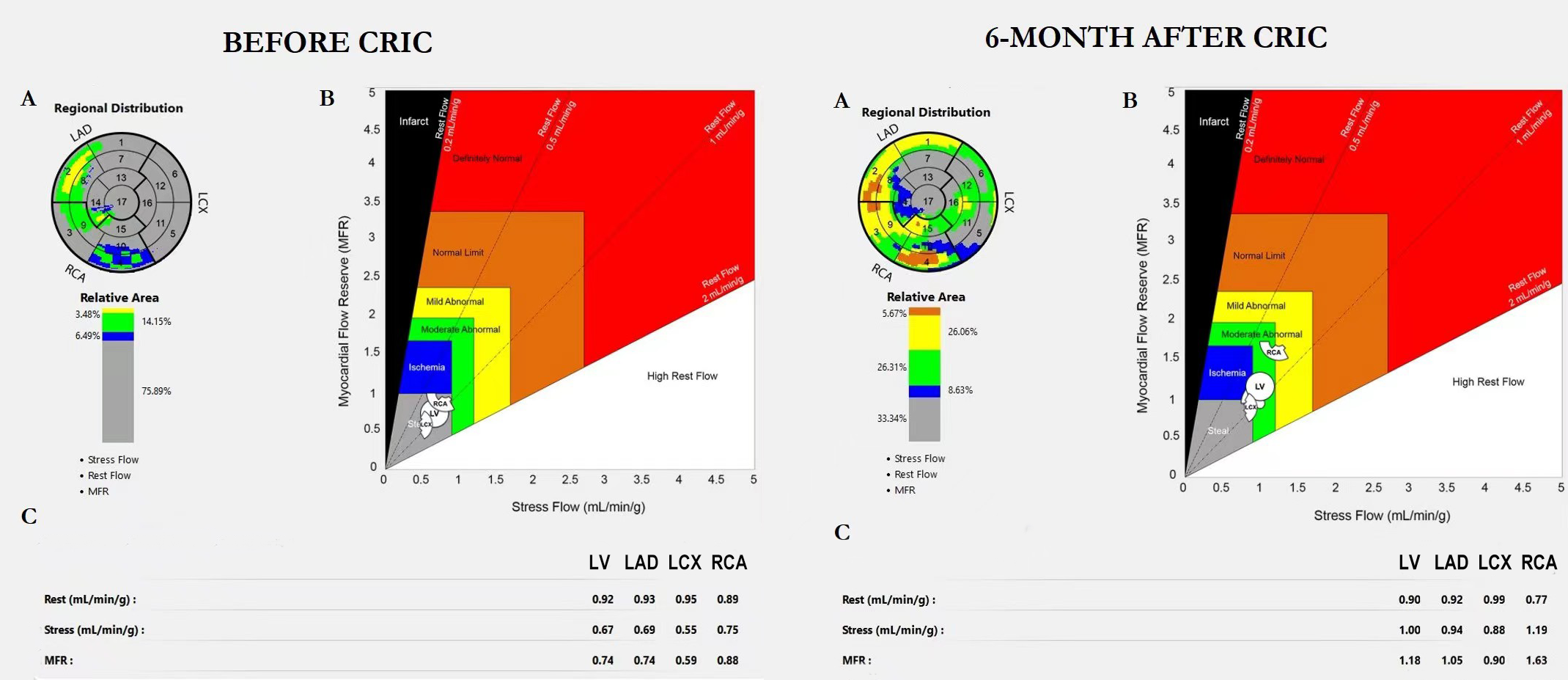

Supplement: Supplementary file 2 — Additional file 2: Figure S1. An example of the myocardial blood flow (MBF) quantification changes presented by single-photon emission computed tomography (SPECT) before and after treatment in a chronic remote ischemic conditioning (CRIC) group patient. Part A: Blood flow status polar map, with a bar graph to represent the extent for each flow status; Part B: The blood flow diagram; Part C: Quantification of rest MBF and stress MBF and myocardial flow reserve (MFR) globally and in individual parts of the left ventricle. [file 12916_2023_3041_MOESM2_ESM.png]

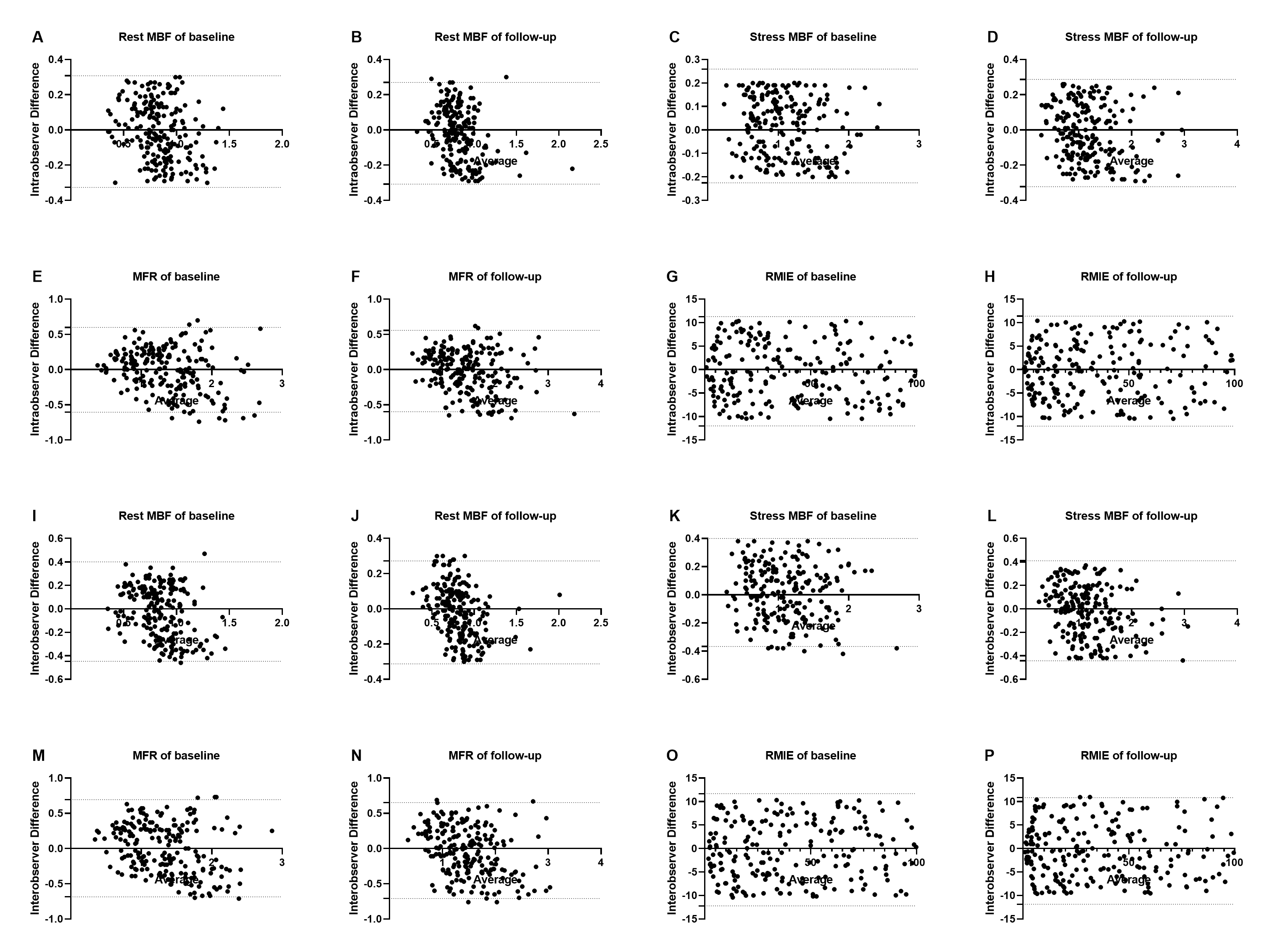

Supplement: Supplementary file 4 — Additional file 4: Figure S2. Differences in repeated measurements of single-photon emission computed tomography outcomes for intra-observer analysis (A to H) and inter-observer analysis (I to P). The dashed lines indicate 95% confidence limits. The results show good agreement across all indicators. [file 12916_2023_3041_MOESM4_ESM.tif]
