# Supplementary material for: Chronic remote ischemic conditioning treatment in patients with chronic stable angina (EARLY-MYO-CSA): a randomized, controlled proof-of-concept trial
Source: BMC Med. 2023 Aug 25;21:324. doi: 10.1186/s12916-023-03041-z (PMC10463998; doi:10.1186/s12916-023-03041-z)
Supplement: Supplementary file 3 — Additional file 3. Supplementary methods included Angina pectoris assessment and Power analysis equation. [file 12916_2023_3041_MOESM3_ESM.docx]

**Angina pectoris assessment**

Canadian Cardiovascular Society (CCS) classification:

There are four levels of severity on the CCS angina classification that are used to assess and grade physical-activity symptoms:

Class I: general physical activity (e.g., walking and stair climbing) is not limited, but angina occurs during strenuous activity;

Class II: Mild limitation of general physical activity. Angina occurs during fast walking, after a meal, in cold or windy conditions, or under mental stress. General limitation of walking more than 200 m on level ground or climbing more than one floor;

Class III: general physical activity is obviously limited, generally walking on flat ground within 200 m, or climbing one floor causes angina;

Class IV: angina can occur during light activity or rest

Seattle Angina Questionnaire：

1. The degree of limitation of each of the following due to chest pain, tightness in the chest and angina pectoris in the past 4 weeks:

|  | Heavily Restricted | Moderately restricted | Mildly restricted | Slightly restricted | Unrestricted | For other reasons Restricted |
| --- | --- | --- | --- | --- | --- | --- |
| Dress yourself |  |  |  |  |  |  |
| Indoor walking |  |  |  |  |  |  |
| Shower |  |  |  |  |  |  |
| Climbing hills or stairs (three levels, non-stop) |  |  |  |  |  |  |
| Outdoor activities or lifting miscellaneous objects |  |  |  |  |  |  |
| A brisk walk  (one kilometer) |  |  |  |  |  |  |
| Jogging (one kilometer) |  |  |  |  |  |  |
| Lifting or moving heavy objects |  |  |  |  |  |  |
| Strenuous exercise  (such as swimming or playing ball) |  |  |  |  |  |  |

2. Episodes of chest pain, tightness in the chest and angina pectoris during maximum intensity activity compared to 4 weeks ago:

A. Significant increase B. Slight increase C. Same D. Slight decrease E. Significant decrease

3. Average number of episodes of chest pain, tightness in the chest and angina pectoris in the past 4 weeks:

A.≥4 episodes/day B.≥1~3 episodes/day C.≥3 episodes/week D.1~2 episodes/week E.<1 episodes/week F. No episodes

4. Average number of nitro medications (e.g., nitroglycerin) for chest pain, tightness in the chest and angina in the past 4 weeks:

A.≥4 episodes/day B.≥1~3 episodes/day C.≥3 episodes/week D.1~2 episodes/week E.<1 episodes/week F. Not used

5. Troublesome compliance with medication due to chest pain, tightness in the chest and angina:

A. Severe B. Moderate C. Slightly D. Minimal E. None F. Not given by physician

6. Satisfaction with various administrations for the treatment of chest pain, tightness in the chest and angina pectoris:

A. Dissatisfied B. Mostly dissatisfied C. Partially satisfied D. Mostly satisfied E. Highly satisfied

7. Satisfaction with the doctor's explanation regarding chest pain, tightness in the chest and angina:

A. Dissatisfied B. Mostly dissatisfied C. Partially satisfied D. Mostly satisfied E. Highly satisfied

8. Overall, satisfaction with current treatment of chest pain, tightness in the chest and angina pectoris:

A. Dissatisfied B. Mostly dissatisfied C. Partially satisfied D. Mostly satisfied E. Highly satisfied

9. The extent to which chest pain, tightness in the chest and angina have affected enjoyment of life in the past 4 weeks:

A. Dissatisfied B. Mostly dissatisfied C. Partially satisfied D. Mostly satisfied E. Highly satisfied

10. How will you feel in your future life if you still have chest pain, tightness in the chest and angina:

A. Dissatisfied B. Mostly dissatisfied C. Partially satisfied D. Mostly satisfied E. Highly satisfied

11. Level of concern about heart attack and sudden death:

A. Always worrying B. Often worrying C. Sometimes worrying D. Rarely worrying E. Never worrying

**Sample Size**

**Power analysis equation:**

1. Find *t_1−α_* based on the central-t distribution with degrees of freedom:

$$df=\frac{{(\frac{\sigma_{1}^{2}}{n_{1}}+\frac{\sigma_{2}^{2}}{n_{2}})}^{2}}{\frac{1}{n_{1}-1}{(\frac{\sigma_{1}^{2}}{n_{1}})}^{2}+\frac{1}{n_{2}-1}{(\frac{\sigma_{2}^{2}}{n_{2}})}^{2}}.$$

2. Calculate the non-centrality parameter:

$$\lambda=\frac{\delta}{\sqrt{\frac{\sigma_{1}^{2}}{n_{I}}+\frac{\sigma_{2}^{2}}{n_{2}}}}.$$

3. Calculate the power as the probability that the test statistic *t* is greater than *t_1−α_* under the non-central-*t* distribution with non-centrality parameter *λ*:

$$Power=\Pr_{Non-central-t}(t>t_{1-\alpha}\mid df,\lambda).$$
