# Supplementary material for: Chronic remote ischemic conditioning treatment in patients with chronic stable angina (EARLY-MYO-CSA): a randomized, controlled proof-of-concept trial
Source: BMC Med. 2023 Aug 25;21:324. doi: 10.1186/s12916-023-03041-z (PMC10463998; doi:10.1186/s12916-023-03041-z)
Supplement: Supplementary file 5 — Additional file 5: Table S1. Intra- and Inter-observer variability in SPECT outcomes. [file 12916_2023_3041_MOESM5_ESM.docx]

Table S1: Intra- and Inter-observer variability in SPECT outcomes

| Intra-observer(n=208) | Measurement 1 | Measurement 2 | ICC | COV (%) | Bland-Altman analysis | | |
| --- | --- | --- | --- | --- | --- | --- | --- |
|  |  |  |  |  | *P* | Bias | 95% limits  of agreement |
| Rest MBF of baseline | 0.83 ± 0.21 | 0.84 ± 0.25 | 0.86 | 13.67 | 0.412 | -0.01 | -0.33 to 0.31 |
| Rest MBF of follow-up | 0.81 ± 0.20 | 0.83 ± 0.25 | 0.87 | 12.83 | 0.062 | -0.02 | -0.31 to 0.27 |
| Stress MBF of baseline | 1.10 ± 0.44 | 1.09 ± 0.46 | 0.98 | 8.04 | 0.051 | -0.02 | -0.23 to 0.26 |
| Stress MBF of follow-up | 1.17 ± 0.49 | 1.18 ± 0.51 | 0.97 | 9.39 | 0.104 | -0.02 | -0.32 to 0.29 |
| MFR of baseline | 1.34 ± 0.46 | 1.34 ± 0.54 | 0.90 | 16.17 | 0.896 | 0.00 | -0.60 to 0.60 |
| MFR of follow-up | 1.45 ± 0.50 | 1.47 ± 0.58 | 0.92 | 14.26 | 0.320 | -0.02 | -0.60 to 0.56 |
| RMIE of baseline | 39.52 ± 28.08 | 39.88 ± 28.71 | 0.99 | 10.57 | 0.389 | -0.36 | -11.99 to 11.28 |
| RMIE of follow-up | 34.69 ± 27.63 | 35.01 ± 27.35 | 0.98 | 12.11 | 0.438 | -0.32 | -12.03 to 11.39 |
| Inter-observer(n=208) | Measurement 1 | Measurement 3 | ICC | COV (%) | Bland-Altman analysis | | |
|  |  |  |  |  | *P* | Bias | 95% limits  of agreement |
| Rest MBF of baseline | 0.83 ± 0.21 | 0.85 ± 0.26 | 0.74 | 18.18 | 0.124 | -0.02 | -0.45 to 0.40 |
| Rest MBF of follow-up | 0.81 ± 0.20 | 0.83 ± 0.25 | 0.88 | 12.98 | 0.054 | -0.02 | -0.31 to 0.27 |
| Stress MBF of baseline | 1.10 ± 0.44 | 1.09 ± 0.46 | 0.95 | 12.64 | 0.226 | 0.02 | -0.41 to 0.40 |
| Stress MBF of follow-up | 1.17 ± 0.49 | 1.18 ± 0.54 | 0.95 | 13.09 | 0.276 | -0.02 | -0.44 to 0.41 |
| MFR of baseline | 1.34 ± 0.46 | 1.33 ± 0.56 | 0.87 | 18.59 | 0.853 | 0.01 | -0.69 to 0.70 |
| MFR of follow-up | 1.45 ± 0.50 | 1.48 ± 0.61 | 0.89 | 16.77 | 0.242 | -0.03 | -0.71 to 0.65 |
| RMIE of baseline | 39.52 ± 28.08 | 39.78 ± 27.78 | 0.99 | 10.86 | 0.536 | -0.26 | -12.22 to 11.70 |
| RMIE of follow-up | 34.69 ± 27.63 | 35.20 ± 27.81 | 0.99 | 11.73 | 0.211 | -0.50 | -11.85 to 10.84 |

MFR: myocardial flow reserve; MBF: myocardial blood flow; RMIE: reversible myocardial ischemia extent; ICC: intraclass correlation coefficient; COV: coefficient of variation; Measurement 1 and Measurement 2 represent repeat measurements by the first observer, and Measurement 3 represents the second nuclear medicine reader who had participated in the initial analysis.
